# Single-Implant Overdentures Retained by a Novel Attachment: A Mixed Methods Crossover Randomized Clinical Trial

**DOI:** 10.1177/23800844221124083

**Published:** 2022-09-20

**Authors:** R.F. de Souza, A.A. Jabbar, D. Jafarpour, C. Bedos, S. Esfandiari, N.M. Makhoul, D. Dagdeviren, S. Abi Nader, J.S. Feine

**Affiliations:** 1 Faculty of Dental Medicine and Oral Health Sciences, McGill University, Montreal, Canada; 2 Faculty of Dentistry, Université de Montréal, Montreal, Canada

**Keywords:** complete denture, dental care for aged, implant-supported dental prosthesis, minimally invasive surgical procedures, patient outcome assessment, removable prosthodontics

## Abstract

**Introduction:**

Single-implant mandibular overdentures (SIMOs) are one of the least invasive implant treatments for edentulism. The new Novaloc attachment system can improve the clinical performance of implant-retained overdentures but has not been tested for SIMOs.

**Objectives:**

To compare Novaloc and a gold standard system (Locator) for SIMOs in an edentate elderly population in terms of patient-reported outcomes and device- and treatment-related complications.

**Methods:**

In this single-center crossover randomized clinical trial (RCT), 10 edentulous participants received an implant in the lower midline and had their lower complete dentures converted to SIMOs. The participants received each attachment system for 3 mo in a randomized order, followed by measurement of patient satisfaction and oral health–related quality of life via the McGill Denture Satisfaction Questionnaire and the Oral Health Impact Profile for Edentulous People questionnaire, respectively. Complications were registered throughout the RCT. Patients were interviewed for their experiences with SIMOs and preference for one of the attachment systems. Quantitative analysis employed mixed linear models and chi-square tests (α = 0.05), whereas interview data underwent thematic analysis and, in turn, integration into quantitative data (mixed methods explanatory design).

**Results:**

All 10 randomized participants completed the trial. Mean ± SD general satisfaction was 92% ± 8% with Novaloc versus 85% ± 13% with Locator (mean difference, 9%; 95% CI, 1% to 17%). For specific McGill Denture Satisfaction Questionnaire items, only denture stability was significantly increased for Novaloc. Seven participants preferred Novaloc over Locator at the end of the RCT (chi-square, *P* = 0.045). No difference was found between the attachments in terms of oral health–related quality of life based on the Oral Health Impact Profile for Edentulous People and complications. Thematic analysis revealed high patient satisfaction with SIMOs, with denture stability the main criterion for their satisfaction and attachment preference.

**Conclusion:**

Among elderly edentulous patients wearing SIMOs, Novaloc led to increased patient satisfaction and preference. Better patient-perceived denture stability may explain this result. The attachment systems exhibited similar short-term maintenance needs.

**Trial Registration:**

ClinicalTrials.gov: NCT03126942 (first registered on April 13, 2017). Secondary identifiers: A03-M07-17A (McGill University, Institutional Review Board) and 2018-3873 (McGill University Health Centre, Research Ethics Board).

**Knowledge Transfer Statement:**

The results of this mixed methods study can be used by clinicians when choosing which attachment system to use for SIMOs. Results suggest that edentulous patients prefer attachments with a better-defined seating position, such as that of the Novaloc system, as opposed to the nylon matrix on metallic abutment of the Locator system.

## Introduction

Complete edentulism is still widely prevalent globally, mainly among the elderly (Muller et al. 2007). Edentulous individuals experience poorer oral health–related quality of life (OHRQoL), mostly due to reduced function and aesthetic changes (Naik and Pai 2011). Specifically for the elderly, the impact of edentulism may include poorer systemic health and even a shorter life span (Holm-Pedersen et al. 2008).

Common restorative practices for the edentulous elderly involve the provision of complete dentures to provide function and quality of life. Conventional dentures are the most common prostheses, but they are not effective in many cases. Several denture wearers are functionally limited and experience significant physical and psychosocial discomfort, even with clinically adequate dentures (Cunha et al. 2013). The mandibular denture is the most common source of these functional problems, given the anatomy of the supporting tissues. Thus, the rehabilitation of edentulous mandibles is one of the most important applications of dental implants (Awad et al. 2000).

Mandibular implant overdentures are probably the most efficient alternative to overcome the functional limitations of treatment with complete dentures. Treatment with a minimum number of implants (i.e., 2 in the anterior ridge) has been promoted by international consensuses, given its major impact on patient-reported outcomes combined with reduced costs (Feine et al. 2002; Thomason et al. 2009). For example, a 2-implant overdenture is a much more cost-effective alternative for the edentulous mandible than a 4-implant fixed prosthesis (Hartmann et al. 2020).

More recently, studies have described the successful use of a single implant to retain a mandibular overdenture (Liddelow and Henry 2010; Harder et al. 2011; Kronstrom et al. 2014; Bryant et al. 2015). When compared with the widely accepted 2-implant alternative, the single-implant mandibular overdenture (SIMO) is able to further reduce treatment cost and time (Hartmann et al. 2020). The treatments are similar in terms of patient satisfaction, OHRQoL (Kronstrom et al. 2014; Bryant et al. 2015), and implant survival rates (Srinivasan et al. 2016). Additional benefits of a SIMO include lower morbidity (e.g., postoperative pain) that tends to be proportional to the number of implants (Ribeiro et al. 2015).

A downside of SIMOs is their dependence on a single attachment for retention/stability, typically comprising cylindrical patrices or O-balls (Liddelow and Henry 2010; Alsabeeha et al. 2011; Harder et al. 2011; Kronstrom et al. 2014; Bryant et al. 2015). More than 50% of SIMO wearers need maintenance of their attachments over the first year of wearing, including reactivation or change of components (Bryant et al. 2015). This is a clinically relevant factor since the loss of retention from worn attachments is a major cause of patient dissatisfaction. Individuals wearing overdentures tend to notice retention loss over time, thus reducing their satisfaction and sense of well-being (Pisani et al. 2017).

Different constituent materials, surface features, and designs can minimize the maintenance needs of attachments (Alsabeeha et al. 2011) and improve patient satisfaction (Bilhan et al. 2011). Novaloc (NL), a recently developed attachment system, has the potential to reduce the patient concerns and maintenance needs of SIMOs. The NL system combines polyetheretherketone (PEEK) matrices and amorphous diamond–like carbon–coated cylindrical abutments, both intended to enhance wear resistance. When compared with the Locator (LC) system, NL shows better resistance to retention loss caused by insertion-removal cycles (Arnold et al. 2020; Wichmann et al. 2020).

The potential advantages of the NL system for SIMOs deserve investigation in randomized clinical trials (RCTs). To our knowledge, no RCT on the NL system has been published, as confirmed by the following PubMed search strategy (updated June 21, 2022): [overdenture* AND (PEEK OR polyetheretherketone OR “Polyether ether ketone” OR “poly-ether-ether-ketone”)].

This article reports the results from a mixed methods crossover RCT comparing the efficacy of NL and LC attachments as retainers for SIMOs of edentulous elders (de Souza et al. 2018). For the quantitative component, the primary outcome variable was patient-reported general satisfaction with mandibular dentures after 3 mo of using each attachment system. We registered other patient-reported outcome variables as secondary/explanatory data: specific satisfaction aspects (e.g., retention/stability), OHRQoL, and choice of attachment. Secondary outcomes involved clinical events, including complications and maintenance needs. The qualitative component aimed to understand the reasons behind patient satisfaction and preferences with SIMOs and attachments, thereby explaining the quantitative findings.

## Methods

We conducted a superiority mixed methods crossover RCT to compare the NL and LC attachment systems with a single implant located in the mandibular midline and used to retain an overdenture. The entire crossover period lasted 6 mo, with participants wearing each attachment for 3 mo. As explained in our published protocol (de Souza et al. 2018), a washout period was not included due to the unlikelihood of a carryover effect.

This article describes the results obtained through 2 methodological approaches: 1) a quantitative approach, based on the statistical analysis of patient-reported outcomes and clinical events during the crossover; 2) a qualitative approach, based on the results of face-to-face interviews about patient preferences and perspectives at the end of the trial. Both methodological approaches were integrated through an explanatory mixed methods design; specifically, qualitative methods were conducted after quantitative analysis and aimed to explain the latter. Although initially planned, a longer follow-up period (12 mo after the crossover) involving a larger sample size was halted due to the SARS-CoV-2 pandemic. This article was prepared according to the CONSORT statement extensions for nonpharmacologic treatment and patient-reported outcomes (Boutron et al. 2008; Calvert et al. 2013).

### Setting

This RCT was carried out at 2 sites in Montréal, Canada. Prescreening, prosthodontic care and maintenance, and data assessment and analysis took place at the coordinating center (Faculty of Dental Medicine and Oral Health Sciences, McGill University). Other steps (screening, implant insertion, and short-term postoperative care) were completed at the Department of Dentistry and Oral and Maxillofacial Surgery at the Montreal General Hospital/McGill University Health Centre.

### Ethical Aspects

This trial was approved by the review boards of McGill University (A03-M07-17A) and the McGill University Health Center (2018-3873). Participants received an explanation of the study goals during the first contact by telephone and were invited to provide written informed consent following an in-person information-and-screening session.

### Recruitment and Screening

Potential participants became aware of this RCT by 1) announcements published in newspapers and magazines for the elderly population and 2) contacting former patients of our university dental clinics who received maxillary and mandibular complete dentures. Information provided by both approaches included a lay summary of the trial and the contact information of a research assistant who could provide more study information and schedule the screening appointment as well as further sessions.

Screening appointments involved an in-depth explanation of the study by 2 researchers (A.A.J., R.F.d.S.) to each potential participant. The explanation described procedures and their potential benefits/risks in addition to the timeline of the trial. A screening form was completed to indicate compliance with inclusion and exclusion criteria. A copy of the trial consent form was provided to each participant at the end of the screening appointment.

Potential participants were invited to undergo cone beam computed tomography (CBCT) scans (I-CAT FLX; Imaging Science International) if considered eligible in terms of their history and clinical examination. The final result of the eligibility of each potential participant was determined after the screening checklist form, medical history, and CBCT image were reviewed. This was performed by the 2 specialists responsible for carrying out the trial interventions (oral and maxillofacial surgeon, N.M.M.; prosthodontist, R.F.d.S.).

### Eligibility Criteria

Inclusion criteria involved being at least 65 y old and completely without teeth for at least 6 mo. Potential participants also had to agree to receive implant stabilization of their lower complete dentures. Medical history could not contraindicate minor oral surgery (i.e., no uncontrolled systemic diseases). The clinical examination had to indicate that there was enough space in the mandibular midline to place a dental implant of 3.3-mm diameter and the ability of the participant to maintain good oral and denture hygiene. Understanding spoken and written English or French and signing the written informed consent were also inclusion criteria.

Inclusion was conditional on the presence of acceptable maxillary and mandibular complete dentures (no fractured tooth or base, minimum tooth wear or none, adequate occlusal vertical dimension, and adequate peripheral extension). Potential participants with unsatisfactory dentures would need to receive adjustments or new dentures before inclusion into the study.

Exclusion criteria were verified by clinical and imaging examination. An exclusion would occur if the potential participant reported any serious medical condition demanding frequent hospitalization, evident cognitive impairment, radiation therapy in the orofacial region, or conditions that may jeopardize the treatment (e.g., alcoholism or smoking >10 cigarettes daily). Any history of implant treatment, as well as acute or chronic symptoms of parafunctional or temporomandibular joint disorder, would lead to exclusion. Potential participants were also excluded if they were unable to participate in our planned study follow-up schedule.

Finally, the imaging examination led to exclusion if the following were observed: areas suggestive of bone lesions, mandibular midline with <11 mm of vertical bone height, insufficient bone width needed to fit the proposed implant, evident endosseous vascular structures in the planned surgical site (Kalpidis and Setayesh 2004), or mandibular ridges graded class I or II by Cawood and Howell (1988).

### Allocation and Blinding

Participants received the abutments according to a sequence determined by computer-generated random coding (simple randomization, 1:1 ratio). Forms with codes were concealed in opaque sealed envelopes, both prepared by a research assistant who was not involved in patient selection and allocation, intervention, and data collection. The codes were stratified by mandibular ridge morphology according to Cawood and Howell, as class III (favorable) versus other classes (unfavorable); however, no ridge was classified as class III. Envelopes were opened only at the appointment when the clinician inserted the first attachment.

Although it was impossible to blind participants and care providers, outcome assessors were unaware of the attachment used by participants, whenever applicable. This was possible for all patient-reported outcome measures, which were collected by a single researcher (A.A.J.), whereas another team member (R.F.d.S.) collected clinical data. Moreover, participants received no information about the expected performance of any of the attachments used. Research team members did not tell participants which was the intervention (NL) or control (LC), even if they could see differences in color and shape (no participant requested this specific information during the trial). Patients also had their appointments scheduled in such a way to minimize their communication with other participants (i.e., a maximum of 1 participant at a time in the waiting room).

### Implant Insertion

Each participant received a single TiZr implant in the mandibular symphyseal region (standard tissue-level implant; Roxolid SLActive, Institut Straumann AG), following the operative procedures recommended by the manufacturer (“Straumann Dental Implant System” 2021). These implants had narrow diameters (3.3 mm) and 2 possible lengths (10 or 12 mm), depending on the patient’s bone structure. The 2 dental specialists (N.M.M. and R.F.d.S.) reached a consensus on implant positioning for all participants according to clinical and radiographic findings. This included a centralized position of the implant cervical surface, avoidance of apical and lateral anchorage in cortical bone, and minimum thickness of denture base material around the attachments (>1 mm).

All participants received 2 g of amoxicillin by mouth 1 h before implant surgery (Esposito et al. 2013). Following insertion, the implants were fitted with healing abutments, and denture bases were adjusted to avoid contact on the surgical site and/or the implant. Participants received standard postoperative instructions and care, including a second appointment after 14 d. At that time, the hollowed portion of lower dentures was relined with a polyvinyl siloxane–based material (Sofreliner Tough M; Tokuyama Dental Corp.).

### Tested Attachment Systems

Participants received their first attachments after 8 wk of implant healing. Both tested attachment systems comprise a polymeric insert in the denture base, which engages a cylindrical abutment (just straight abutments were used in this study). The NL attachment system (Institut Straumann AG) is composed of amorphous diamond-like carbon–coated NL abutments, PEEK inserts, and titanium housings. Different levels of retention are possible with different PEEK inserts, coded by color. To achieve a standard retention level, this trial used only the medium retentive inserts (yellow). The comparator attachment system (LC system; Zest Anchors, Inc.) uses nylon inserts and TiN-coated abutments. For the LC, just the medium retention inserts were used (pink). Mean ± SD initial retention forces for tested systems were 1.0 ± 0.1 kgf for NL and 3.7 ± 0.8 kgf for LC, according to our unpublished in vitro data (*n* = 10 per system).

For both systems, the abutment cuffs should be 1 mm higher than the peri-implant mucosa. The lower dentures were checked for fit and occlusion and adjusted as needed with the metallic housing over the abutment. Once no contact between the attachment parts and denture was ensured, a chairside retrofit procedure was carried out with a self-curing hard relining acrylic material (GC Reline Hard; GC America) (“Restoring Straumann” 2014). For each system, the tools recommended by the manufacturer were used to handle abutments and inserts. After occlusal adjustment, finishing, and polishing, participants received denture hygiene instructions.

The first attachment was worn for 3 mo and changed after that period. The attachment change was facilitated by carefully grinding the relining material to loosen previous metallic housings. After abutments and housings were removed, the same retrofit procedure was carried out with a different attachment. As with the first attachment, the second was worn for 3 mo, resulting in a total 6-mo follow-up period. Participants kept the attachment of their preference at the end of the follow-up period.

### Outcomes

Outcome data collection took place 3 times throughout the RCT: at baseline and after 3 mo of wearing each attachment. Baseline data collection included sociodemographic and clinical data taken before implant placement surgery.

At the 3 data collection points, participants rated their satisfaction with their lower dentures through the McGill Denture Satisfaction Questionnaire (MDSQ; Awad and Feine 1998; de Souza et al. 2020). The MDSQ is composed of 8 core questions, including general satisfaction (primary outcome) and specific aspects (chewing ability, comfort, stability, appearance, speech, and ability to clean). An additional 14 questions refer to the individual’s perceived ability to chew specific types of food. Participants rated their satisfaction with all items on a 100-mm visual analog scale (VAS). Anchor words placed at 0 and 100 mm represented “no satisfaction at all” and “complete satisfaction,” respectively. At each of the 3 appointments, participants were reminded to answer the items on the MDSQ with a VAS training questionnaire, which contained “0%” and “100%” as anchor points. An additional question about the perceived rotation of overdentures was included at the end of each 3-mo follow-up, per our published protocol (de Souza et al. 2018). However, the number of nonrespondents (>20%) and participants who answered incorrectly (e.g., open-ended answers on a VAS) precluded us from using these data.

We collected data on OHRQoL before and after each attachment using the Oral Health Impact Profile for Edentulous People (OHIP-EDENT) questionnaire (Allen and Locker 2002). The version used in this RCT generates a summary score from 0 to 120, with better OHRQoL represented by higher values. Questions were grouped according to 4 subscales, as recommended for edentulous individuals (Souza et al. 2010): masticatory-related complaints, psychological discomfort and disability, social disability, and oral pain and discomfort.

At the end of the study, we asked participants to choose their preferred attachment: the first, the second, or no preference. Participants had the option of replacing the second attachment with the first, if that was their preference. This question was presented by a team member uninvolved in previous trial stages (C.B.), who was unaware of the attachment in use.

This study included clinician-reported outcomes during the period between each attachment insertion and follow-up. Overdentures and implants were classified as “successful” or “surviving” after each 3-mo follow-up, as proposed by Misch et al. (2008). We also examined device- and treatment-related complications, such as oral pain/soreness, damaged or replaced attachment components, damaged upper and lower dentures, presence of calculus and plaque accumulation, as well as signs of peri-implant disease. Any complication noted by participants or observed through clinical examination was reported.

### Trial Interruption and Sample Size

This trial was interrupted due to the COVID-19 pandemic, which prohibited research activity requiring physical contact with participants between March 2020 and June 2021. Thus, we ran an interim analysis to determine whether continuing the trial would be feasible to achieve our originally planned sample size of at least 21 participants (alpha = 0.01, power = 90%). Since the available sample of 10 patients allowed for the detection of significant differences in the primary outcome, we decided to terminate patient recruitment and followed up with the patients already recruited.

### Statistical Analysis

Each attachment was compared with data for the separate questions of the MDSQ and the summary scores for the OHIP-EDENT. In addition to descriptive analysis, the mean differences and 95% CIs were used to compare the effect of the tested interventions. A mixed linear model was used to test for a carryover effect of the primary outcome (general satisfaction with lower overdenture): the model included the effect of attachments and time (3 vs 6 mo), as well their interaction. The chi-square test was used to assess patient preference toward one attachment system. Descriptive statistics were used to analyze complications, and if applicable, comparisons were performed by using the sign test. Statistical tests were performed with SPSS 21.0 (IBM) with α = 0.05.

### Qualitative Assessment

Following the quantitative phase, we carried out a qualitative descriptive assessment (Sandelowski and Barroso 2003) to obtain an in-depth understanding of 1) patient experiences with SIMOs, 2) rationales for preferring a particular attachment, as well as 3) insight on the advantages and disadvantages of each system.

Nine patients participated in individual semistructured interviews, all in French. The interviews were held in a quiet meeting room outside the clinics to ensure a confidential discussion. To follow an explanatory mixed methods strategy, an interview guide was created on the basis of quantitative data collection forms to further explain those findings. An experienced researcher (C.B.) steered the interviews using the guide, which included open-ended questions regarding participants’ history, their expectations and experiences with their SIMOs, their perceptions of each attachment, and the reason for their preferences. The guide also approached the subject of the 8 core questions of the MDSQ as open-ended questions.

Each interview was audio recorded and transcribed verbatim, followed by a qualitative thematic analysis, which is an exploratory method of recognizing and reporting patterns, known as themes, within the collected data (Braun and Clarke 2006). Coding of the transcripts was performed with the MAXQDA Standard 12 (version 12.2.1; VERBI GmbH). We initiated the analysis by adopting a deductive coding strategy based on the interview questions and the theoretical framework of satisfaction for the themes covered by MDSQ items (de Souza et al. 2020). After going through the transcripts, we proceeded with an iterative inductive analysis to add any emerging codes that reinforced our initial coding structure. Then, the codes and subcodes were categorized together by their commonalities. Finally, the emerging themes were identified, and the relevant passages were extracted from the transcribed interviews.

## Results

### Quantitative Phase

#### Screening and Participant Flow

Thirty-eight individuals underwent screening appointments. Most were screened from the winter to the summer of 2018, with a 35% success rate from advertisements in seniors’ magazines. Subsequent screening efforts continued until spring 2019 and involved contacting former patients; this resulted in the final recruitment of 17 participants.

The participant flow from screening to final data analyses is presented in the Figure. Reasons for noneligibility during screening were systemic disease, maintenance costs, inability to return for recall visits, a history of radiotherapy, heavy smoking, and rejecting implant therapy (Table 1). Three potentially eligible individuals did not provide consent and thus were not included, while another 4 were excluded due to insufficient ridge height observed through CBCT examination. The RCT initially involved 11 participants, of which 10 completed the 6-mo crossover (5 per sequence).

**Figure.**
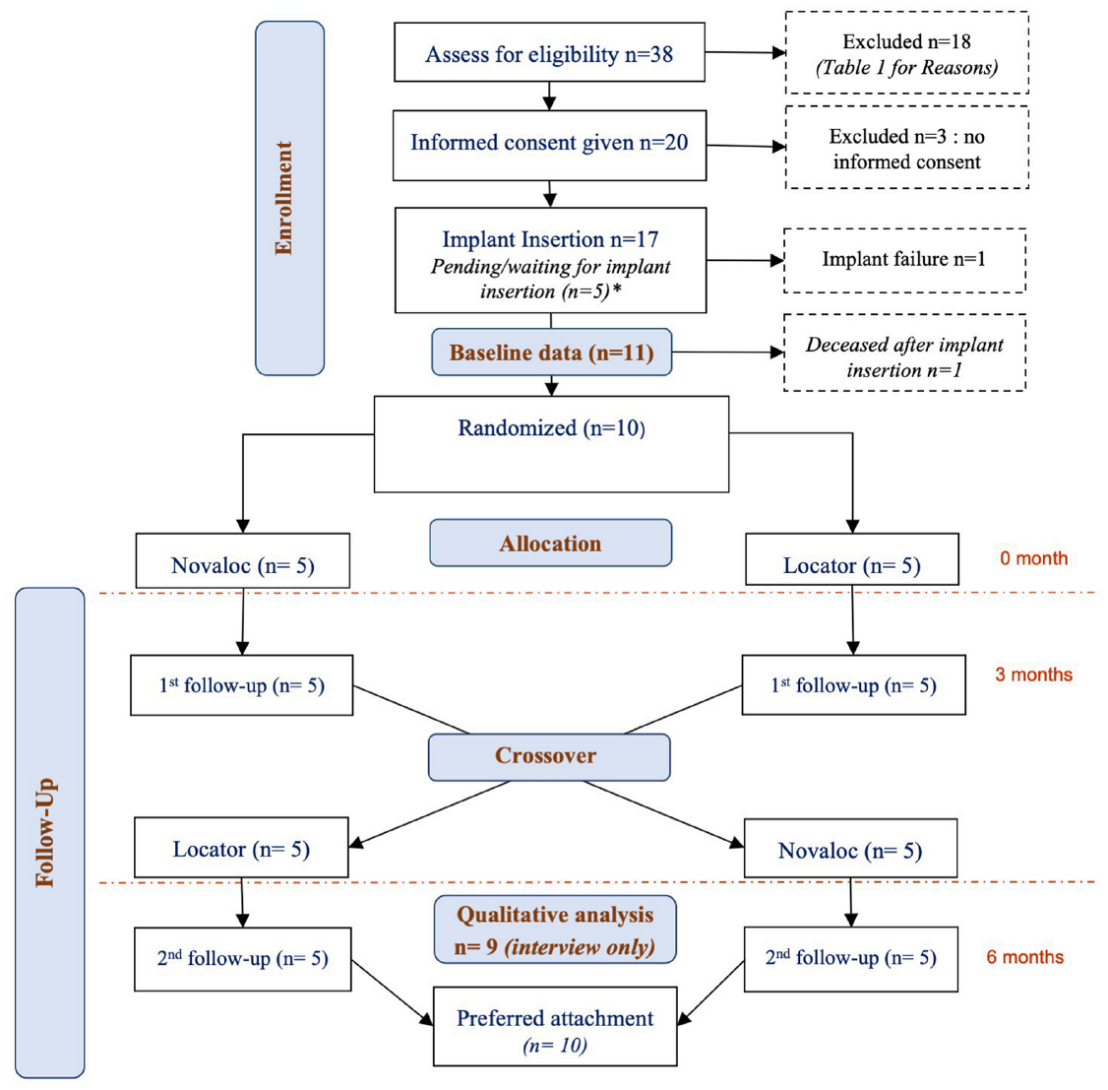
CONSORT study flowchart (* Implants not placed due to trial interruption).

**Table 1.** Reasons for Exclusion of Participants after Screening.

Reasons for Exclusion	*n*
Systemic conditions contraindicating minor oral surgery	5
Vertical bone height <11 mm (assessed by CBCT)	4
Unable to return for study visits	3
Refused implant stabilization (a): fear of surgery	3
Refused implant stabilization (b): declined due to the cost of maintenance	2
History of radiotherapy	2
Refused implant stabilization (c): did not want to adjust the dentures	1
Heavy smoker	1
Refused implant stabilization (d): refused to receive an implant	1
Lost contact after screening/before implant placement	1

Some participants are listed in >1 category because they had multiple reasons for exclusion.

CBCT, cone beam computed tomography

No participant withdrew from the trial or requested any modification in the interventions. All 7 nonevaluated participants were lost due to reasons unrelated to the tested attachments: 5 were enrolled shortly before the onset of the COVID-19 pandemic and could not receive the intervention; 1 participant had a failed implant before attachment insertion that could not be replaced before the pandemic; and 1 participant received a diagnosis of glioblastoma between implant placement and attachment insertion and passed away shortly thereafter.

#### Characteristics of the Sample

Our randomized sample consisted of 6 women and 4 men, with a mean age of 68 ± 2.5 y. The lower ridges of 4 participants were classified as class IV by Cawood and Howell (1988), 5 as class V, and 1 as class VI.

At baseline, ratings of general satisfaction in both groups were not significantly different. Differences were not significant for most of the other questions in the MDSQ, with the exception of “ease of cleaning” and “ability to chew hard cheese.” Refer to Appendix Table 1 for detailed outcome data on the baseline. Of 10 participants, 4 answered “yes” when asked if their oral condition affected their general health, and 6 answered “no.” Reasons for an affirmative answer were 1) limitations in food choices (i.e., less fresh vegetables and fruits) and 2) difficulty digesting due to improper chewing. Regarding OHRQoL scores, the mean value of the participants who received NL first was 77 ± 24, as opposed to 95 ± 21 with LC. The mean difference (−18; 95% CI, −51 to 15) was not significant.

#### Postintervention Data

Posttreatment values obtained for patient satisfaction with SIMOs can be seen in Table 2. Ratings of general satisfaction (primary outcome) were significantly higher for NL than for LC. For the specific items, similar results were found for denture stability ratings, with a significantly increased value for NL. Other items (cleaning, speech, comfort, and aesthetics) reached similar results between attachments. Analysis of carryover effects in general satisfaction showed no significant impact for follow-up time and attachment-time interaction (mixed linear model: *P* = 0.551 and *P* = 0.522, respectively).

**Table 2.** Posttreatment Values for the MDSQ and OHIP-EDENT according to Each Attachment and Between-Treatment Differences.

	Attachment, Mean ± SD		
Component	Novaloc	Locator	Mean Difference (95% CI)
General satisfaction	92 ± 8	85 ± 13	9 (1 to 17)
MDSQ			
Cleaning	89 ± 17	88 ± 9	2 (−9 to 12)
Speech	94 ± 7	94 ± 5	1 (−2 to 5)
Comfort	91 ± 7	84 ± 13	7 (−5 to 19)
Aesthetics	93 ± 8	91 ± 9	3 (−5 to 10)
Stability	83 ± 6	68 ± 16	16 (4 to 28)
Chewing ability			
General ability	88 ± 5	83 ± 13	7 (−3 to 16)
White bread	90 ± 7	91 ± 13	0 (−9 to 9)
Hard cheese	88 ± 11	79 ± 23	11 (−3 to 25)
Raw carrots	69 ± 29	54 ± 28	12 (−4 to 27)
Salami	71 ± 25	55 ± 28	16 (−2 to 33)
Steak	74 ± 30	76 ± 23	0 (−14 to 13)
Raw apples	74 ± 18	66 ± 28	11 (0 to 22)
Lettuce	88 ± 15	87 ± 12	3 (−5 to 10)
Chewing function			
General function	81 ± 22	87 ± 8	−4 (−20 to 11)
White bread	83 ± 17	86 ± 10	−2 (−11 to 7)
Hard cheese	85 ± 18	84 ± 20	3 (−4 to 10)
Raw carrots	66 ± 32	67 ± 25	0 (−20 to 19)
Salami	66 ± 34	61 ± 25	5 (−10 to 20)
Steak	74 ± 29	72 ± 24	3 (−7 to 14)
Raw apples	77 ± 29	78 ± 25	2 (−14 to 17)
Lettuce	80 ± 27	89 ± 9	−8 (−27 to 10)
Oral condition	88 ± 13	85 ± 12	4 (−7 to 15)
MDSQ domains			
Factor 1: overall satisfaction—lower denture	88 ± 7	83 ± 7	6 (−1 to 13)
Factor 2: denture hygiene and appearance	91 ± 12	90 ± 7	2 (−7 to 11)
Factor 3: mastication-related subset	77 ± 19	76 ± 17	4 (−4 to 11)
OHIP-EDENT, total	108 ± 5	107 ± 6	4 (−2 to 5)
Masticatory-related complaints	20.5 ± 2.3	19.9 ± 1.9	0.6 (−0.5 to 1.7)
Psychological discomfort and disability	27.4 ± 2.0	26.6 ± 1.5	0.8 (−0.9 to 2.6)
Social disability	34.8 ± 1.4	34.9 ± 1.4	−0.1 (−0.9 to 0.7)
Oral pain and discomfort	25.2 ± 2.4	24.7 ± 2.5	0.6 (−0.8 to 1.9)
Unsatisfactory diet	5.6 ± 0.5	5.5 ± 0.5	0.0 (−0.3 to 0.3)

Responses provided on a 100-mm visual analog scale. Differences consider only cases with data for both attachments (*n* = 10).

MDSQ, McGill Denture Satisfaction Questionnaire; OHIP-EDENT, Oral Health Impact Profile for Edentulous People.

Results for OHRQoL are shown in Table 2, and these were similar between attachments. Mean values approached the maximum values of the scales, reflecting good OHRQoL. Data from the separate domains/subscales (e.g., “masticatory-related complaints”) also appear to be similar between attachments.

Seven participants preferred NL at the end of the crossover period, which was the second attachment for 5 of them, whereas 2 requested to have NL (first attachment) back as they had received LC as the second attachment. The other 2 participants preferred LC (second attachment used in both cases), and a single participant was indifferent and kept the last attachment (NL). The hypothesis that preference (NL, *n* = 7; LC, *n* = 2; indifferent, *n* = 1) occurs by chance was rejected (chi-square, *P* = 0.045).

A total of 13 device- and treatment-related complications occurred for the 10 participants who concluded the crossover, with 6 and 7 events attributed to the use of NL and LC, respectively (sign test, *P* = 0.727, nonsignificant). The most common complication was the presence of sore spots under the SIMO following attachment insertion (NL, *n* = 5; LC, *n* = 3), which was resolved by making minor adjustments to the SIMOs. Three participants needed the replacement of plastic retention matrices/inserts (NL, *n* = 1; LC, *n* = 2); all of them presented visibly deformed matrices due to incorrect insertion, which provided minimal retention. There were 2 participants with fractured SIMOs, both with LC.

### Qualitative Phase

Interviews with the 9 participants (P1 to P9; 6 women and 3 men) carried out in this phase unveiled details on their experiences and perceptions, which could be grouped into 2 major themes: 1) how SIMOs differed from their complete standard dentures and 2) differences between the attachments. Table 3 presents quotations from participants that represent those themes and their subdivisions.

**Table 3.** Themes from Qualitative Analysis of Interviews and Representative Quotes for “Comparison between SIMOs and Previous Dentures/Edentulism.”

Stability: general	*P7*: Before, to have the same stability, I used glue [denture adhesive] at the top and at the bottom. . . . Now I don’t use glue anymore. *P9*: Even if it’s only one implant, I still see a big difference and my [new] prosthesis holds up very well. . . . It doesn’t move like it did before.
Stability: impact on oral function	*P1*: When I yawn, well I know that I have to be careful. *P3*: Let’s say you’re going to eat with someone, and your dentures always move in your mouth, it’s embarrassing, you know . . . if you’re talking to someone and then the dentures move in your mouth. . . . It’s not fun *P4*: Sometimes the [previous] prosthesis down there came out when I was talking.
Comfort	*P2*: The more stable it is, the more comfortable it is for you *P5*: . . . I had some [teeth] to chew on but when I smiled you could hardly see, you know, and so it was like empty . . . which was uncomfortable . . . the minute I saw that [SIMO], you know, it was like a feeling of safety. *P9*: [When] there’s less food that goes underneath . . . we get hurt less.
Aesthetics	*P5*: My teeth are visible, before, they were not visible. . . . So you feel more comfortable, it’s less embarrassing and I was afraid, sometimes I felt that people looked at me as if I had no teeth. . . . because my teeth were worn out. . . . So I felt like I was old [*laughs*], you know, or there’s a problem with your teeth or you don’t have any teeth you know *P7*: I feel like I have my natural teeth. *P8*: Sometimes I don’t even realize that it’s there.
Ability to speak	*P1*: When I was talking, the saliva was coming out of my mouth. . . . You see that was annoying too and since I got the implants, I speak much better . . . the prostheses plus the physiotherapy [done for mild discomfort in the neck region], I think I’m practicing, so it’s going to be okay. *P4*: It seems to me that when my lower prosthesis was not glued on, I don’t know how to tell you that, depending on what I was talking about . . . if I was hurrying to speak or if the words we were saying were hissing, well . . . well, my prosthesis was moving.
Ability to chew: improved	*P4*: It’s really a big difference. I can eat anything I want and I tried a subway [sandwich]. . . . It worked on the first try. *P6*: I ate hot dogs not so long ago. I ate them all. I can chew well and it’s better for the intestines anyway. *P7*: I eat and recently we ate a steak that went very well. . . . Pretty much all foods, nuts, nuts are hard to chew, I don’t have any problems [with them] either. . . . So no it’s going well, it’s going well. . . . With the old prosthesis, it was not good. *P8*: I eat apples. I don’t have to cut them. I eat as if I had no dentures *P9*: Then I could eat and chew as I should.
Ability to chew: limitations	*P3*: I can eat almost anything. . . . It’s sure that I still have to slice it thinly here *P6*: Only I can’t eat too much, too hard foods. . . . Because that’s where it’s going to move, and I can even bite my cheek sometimes, I bite my cheek from time to time *P7*: Biting into a hard apple, I ah eat them in slices, I slice the apple there are things that you have to be careful with because I don’t want to damage my teeth either, like trying to crack a nut, a big nut . . . walnuts or something. . . . Uh I’ll take a tool, there are tools for that, I’ll take a tool.
Ability to clean	*P5*: I know how to maintain it, or to rinse it after dinner, you know, all the time. . . . I have no problem with that *P6*: Very very very easy. I clean it every morning I have a denture brush here and then . . . to prosthesis then it goes very, very well. *P8*: Well, it’s like, it’s like . . . my other dentures . . . I’m careful because I don’t rub too hard, it’s like washing our other teeth, you know, we’re careful, we soak them in the same way.

SIMO, single-implant mandibular overdenture.

#### Comparison between SIMOs and Previous Dentures/Edentulism

The participants were generally very satisfied with the SIMO and preferred it over their previous condition (complete denture/edentulism) due to several reasons, such as a huge improvement in denture stability. They reported that their SIMOs were more functional and stable than the conventional denture since it was more firmly seated during function, movement was reduced, and less denture adhesive was required. Stability was the central criterion for choice of denture, based on which participants perceived other aspects of satisfaction (comfort, ability to speak, eat, and clean). The movement and instability of the conventional denture during speaking, eating, and yawning interfered with the participants’ sense of confidence and comfort.

Stability was important for the participants in terms of providing the ability to eat since “the food becomes lodged under the prosthesis” (P2) if the denture is unstable during eating. Constant food beneath their conventional prosthesis disturbed their ability to eat as they felt the urge to stop eating and clean the prosthesis, whereas some of them claimed that this was not an issue with implant-supported overdentures: “I never had food under my implant, never” (P4).

The participants perceived comfort differently. While for some the stability of the denture was synonymous with comfort, others considered aesthetics an important criterion of comfort. For instance, less movement and higher stability of the implant-retained prosthesis during function gave one of the participants a more “comfortable” experience (Table 3, P2). Yet, with denture teeth more apparent on the single-implant overdenture, participants’ comfort was affected and provided a feeling of safety (P5). Furthermore, less food beneath the implant-retained prosthesis made participants feel more comfortable.

Some participants found the SIMO more aesthetic due to better tooth visibility and similarity to natural teeth. As mentioned, when teeth were less visible with the conventional denture, this adversely affected feelings of comfort.

Owing to the instability of the conventional denture, participants cited difficulty in speaking with their previous conventional dentures, which consequently interfered with their feelings of comfort. The participants did not note any issues with speaking with the implant-retained prosthesis. One of them indicated an improvement in speaking with the implants in combination with speech therapy (Table 3, P1).

With regard to the ability to chew, the participants reported having difficulty with their conventional dentures resulting from their instability that in turn led to uncomfortable experiences (P3, “It was embarrassing to try to eat”; P4, “Depending on what you eat, [the denture] would come off”). The participants found an improvement in the ability to chew with their implant-based prosthesis as compared with their previous conventional dentures. They stated that they could eat and chew almost everything with the single-implant overdenture, as if they had their own teeth. Nevertheless, some indicated that they were still careful with food, needing to cut it into pieces and were unable to eat hard and sticky food.

The participants stated that the SIMO was easy to clean. One participant stated that despite taking care to properly clean the implant-retained denture, there is no difference in cleaning between the dentures.

#### Comparison Between NL and LC

Even though participants were generally very satisfied with both attachments, thematic analysis clarified the reasons for their preference of NL over LC. Stability was the main reason behind participant preferences. Most participants indicated that NL was more solidly fixed in the mouth, lasted longer, stayed in place better during function, and was more stable than the LC, though 1 participant found the difference insignificant (Table 4). Another participant suggested that the LC became loose over time: “my second one [LC] was looser, was looser, I put my tongue in and then my, my prosthesis moved a bit. . . . But not at the beginning, it’s strange, but not at the beginning. At the beginning it was perfect, it was very tight, it was perfect and then it’s been more than 3 months, for almost a month that it’s loose” (P4). Only 1 participant noted better stability with the LC than the NL attachment (P3).

**Table 4.** Themes from Qualitative Analysis of Interviews and Representative Quotes for “Comparison between NL and LC.”

Stability: general	**NL > LC** *P1:* Lately often when I yawn or when I eat, it [LC] lifts it up more easily. *P2:* This one [NL] looks like it sticks better. . . . It’s more stable, the magnet, the magnet closes better. *P7:* I remember the old attachment [LC], sometimes I ate certain foods and then I had the impression that the prosthesis came out of the attachment. The only difference is that I think there was perhaps a little less stability with the old one [LC], but it wasn’t marked there, it wasn’t a major difference. *P9:* I found that my prosthesis was more solid, held better in my mouth with this attachment [NL] and also, when I ate, I chewed, it hardly moved. Whereas with the other one [LC], I had had a little bit of problems too . . . well I found that the prosthesis held less well, it moved. **LC > NL** *P3:* My mouth dries easily, and [when] I talked with people, it was chhhhhhhh . . . and then the dentures [NL] started to move and then, come out of the mouth . . . [with LC] I didn’t put any glue [denture adhesive] on it. . . . It doesn’t move. . . . . Now [with LC] I use so little [glue] that my two tubes that I had three months ago are not finished . . . whereas in the first one [NL] I could put one [tube] in every 2 weeks, it was really fast.
Stability: clicking sound	*P2:* You feel the click [with NL] and then it fits together. *P5:* You’ve taken the feeling of safety with the noise. It seems to me that it’s a little better [with NL] because when they put it on it clicks better it seems, it clicks. . . . It seems like it’s tighter. *P7:* When I insert it, it clicks. So you can honestly hear the click to check that it’s good, that the junction is good between the, the, the implant, between the attachment and the prosthesis. [The click] was my way of confirming that it’s installed . . . confirms to me that the prosthesis is going to be stable.
Comfort	*P2:* The edge . . . uh the circle on top . . . it’s, it’s less sharp, that is to say, it doesn’t cut [with NL]. *P4:* It was during certain things when you bite into something stickier, let’s say. . . . Well it [LC] could be uncomfortable. *P7:* It [NL] is efficient, it’s stable and easy to install and it’s also easy to remove because when I take it out, I have to apply a little pressure with my thumbs so that the prosthesis doesn’t come out of the attachment. *P9:* When I was eating, yes, it [NL] almost didn’t move, there was almost no food entering under the prosthesis either.
Aesthetics	*P2:* The color of the, of, of the first implant [LC] it’s a little golden and it’s more pleasant . . . while the current implant [NL] is all black, it’s . . . like a shock. *P4:* I like the dark one.
Ability to chew	**NL > LC** *P1: (When asked, “What can you do with the yellow [NL] that you can’t do with the pink [LC]?”)* Eat faster maybe . . . whereas with the pink one [LC] I have to be a little more careful with my food. I can eat more easily [with NL]. *P7:* [The study prosthodontist] had suggested that I try a different attachment to confirm if it was stronger, and then on chewing it’s stronger. It never happened that my denture came out of the attachment. *P9:* I even ate corn and there was no problem. . . . Let’s say eating bread . . . it was better with the second one [NL]. It was always better with the second attachment, yeah. **LC > NL** *P3:* What I bought for myself at the end of the week is Tostitos, I was able to eat them and I wasn’t able to eat them before the meats, in general, the meats I couldn’t even try . . . whereas here, well, I still make sure that it’s sliced thin, but there’s no problem.

LC, Locator; NL, Novaloc.

Most participants placed great importance on the click sound that occurred when the NL overdenture was completely inserted. Participants reported that they could hear the click better with NL, which affected their perception of stability. They regarded the click sound as an essential criterion of stability and claimed that it gave them a sense of safety, showed tightness, and confirmed the proper positioning of the prosthesis. A single participant disregarded the importance of a click sound while inserting the prosthesis: “even if it doesn’t click, we know it’s there because it goes into the mouth and then you touch it with your tongue, so it shouldn’t move” (P8).

Some participants found that NL was more comfortable than LC due to less denture base movement during function (e.g., eating, speaking, and yawning), less sharp edges on the top of the abutment, less food under the prosthesis, and greater ease of wear. Some participants stated that LC was less comfortable than the NL attachment since the prosthesis would lift more easily when eating.

Regarding aesthetics, the color of the abutment was a matter of concern for some participants, even if it was visible only when the SIMO was removed. One of the participants favored the golden color of the LC and disliked the black color of NL. Yet, another participant favored the dark color of NL. For the others, no difference was expressed in aesthetics between the attachments.

The ability to speak was perceived to be similar between attachments for some participants (P9: “Uh, yes, for talking, I can’t say, both were quite similar. . . . I never had any difficulty talking with either of them”). Whereas one participant reported the ability to pronounce words such as “s” and “ch” with NL (P2: “[NL] doesn’t prevent me from pronouncing the ‘se,’ the ‘che’”), another one noted improvement in talking with LC (P3: “there’s an improvement in the, uh, in the way you talk [with LC]”).

Most participants found NL better for chewing than LC. Some reported chewing corn, bread, steak, and nuts with NL. One participant claimed being able to eat faster with NL, whereas careful eating was required with LC (Table 4, P1). However, the participant who indicated greater stability for LC also preferred it over NL in the ability to chew (P3).

Finally, while some found no difference in the ability to clean the 2 attachments, some participants stated that NL was smoother and thus easier to clean (P2: “I found that when I was cleaning . . . I felt that I could not remove the tartar from the implant. . . . But this one [NL] is better, you can feel that it’s smoother. . . . So I prefer the second one [NL]”).

## Discussion

This mixed methods crossover RCT compared NL and LC attachments for retaining SIMOs, with more favorable results for NL. Our sample of edentulous elderly patients reported greater satisfaction when their lower SIMOs were retained with NL, which most of them preferred. NL was comparable to LC in terms of OHRQoL and complications.

General patient satisfaction with lower complete dentures tended to improve greatly during 3 mo with both tested attachments. Moreover, the wide variation in results observed with conventional dentures was mitigated with the use of both attachments. These findings imply that SIMOs reach a more homogeneous patient response, leading to more predictable results in our population of interest. Other studies have demonstrated wide variation in patient satisfaction with conventional dentures that are comparable to our baseline results (Awad and Feine 1998; Stober et al. 2012). Our qualitative data complement the quantitative findings and endorse the huge effect of SIMOs on patient satisfaction, due to benefits in practically all aspects considered by the MDSQ.

Posttreatment satisfaction with SIMOs resulted in significant differences. However, participants tend to be more satisfied in general when treated with NL than LC, with a mean difference close to 10% of the measuring scale. These findings highlight the different performance of tested attachments for SIMOs, which was detectable even with a small sample size of 10 participants. A crossover trial with 12 participants was unable to detect differences between LC and magnets for the same indication (Cheng et al. 2012). Results for preference reinforce differences in general satisfaction with SIMOs. When we asked our participants to choose one attachment to keep, the majority (70%) preferred NL. That trend was significant, even if our sample size did not allow a precise estimate of preference rates. By comparing the thematic analysis of interviews with the quantitative data obtained from the questionnaires, we identified the reasons for their preference. As observed, participants felt that NL and LC perform differently under function and during insertion/removal.

The added insights on specific items of the MDSQ, available through the mixed methods approach, shed light on the reasons for different patient satisfaction with tested attachments. Participants felt that their SIMOs were more stable with NL, even if initial retention forces may be higher with LC. This is not surprising given the materials used in the tested attachments and qualitative output. Two recent in vitro studies compared NL and LC, with the first showing more resistance to wear and less retention loss with NL after compression and insertion-removal cycles (Arnold et al. 2020; Wichmann et al. 2020). This RCT endorses that patient satisfaction with SIMOs and subjective perceptions of denture stability are inversely proportional to retention loss with use, rather than initial retention, as observed with 2-implant overdentures (Pisani et al. 2017). Moreover, current findings show that the differences between tested attachment systems are clinically relevant from a patient perspective. From a qualitative standpoint, it is noteworthy to take subjective factors, such as the perception of the click sound, into account when considering patient satisfaction with SIMOs. According to the participants, the click sound was an important factor behind how they perceived the overdenture stability. The more noticeable click sound with PEEK-based matrices, more rigid than their nylon counterparts, is a major reason behind greater satisfaction with NL than LC.

Other MDSQ items showed no significant difference, which implies that the attachments have a similar influence on other satisfaction-related aspects and masticatory ability. For example, the roughly similar geometry of NL and LC does not interfere with oral hygiene from the patient standpoint. Comfort with SIMOs as well as the patient’s ability to speak tends to be similar regardless of the attachment. Given the nature of the interventions, they also exerted a nonsignificant influence on aesthetics, according to our statistical findings. However, our mixed methods approach added insight to the quantitative data and shed light on the importance of the aesthetics of single-implant overdentures to some of the participants. According to the qualitative analysis, although the implant was visible only when the overdenture was removed, the shape/color of the implant was a matter of concern for some participants, which was quite unexpected. This shows that aesthetics bears not only a social dimension (the beauty of the smile) but also a more intimate one (the aspect of the mouth that is visible just to the individual), which manufacturers could take into account.

Different attachments have no detected significant differences in terms of OHRQoL or self-perception of oral conditions. Both tested attachments led to major improvement, in agreement with well-described results with mandibular implant overdentures (Kodama et al. 2016). These findings can be explained by a wider array of variables behind self-perceived oral health as compared with patient satisfaction with SIMOs (Souza et al. 2010; de Souza et al. 2020). Other studies showed the influence of different attachment types and numbers on OHRQoL by using larger samples (de Souza et al. 2015). Thus, we cannot discard the influence of tested attachments on OHRQoL; however, it is valid to underscore the lower sensitivity of such a construct to the performance of prosthetic components than patient satisfaction. Both tested attachments led to a comparable number of prosthetic complications, all with mild severity. All were clinically manageable and suggested that executing SIMOs with NL or LC will lead to the same routine from the clinician perspective. Moreover, this trial provides evidence that the novel attachment system, NL, is safe and comparable to the LC system, which is a well-known attachment system.

Current results are generalizable to patients with mandibular implant overdentures in general (Vogel 2008). This includes a balanced gender distribution and advanced age, with mild to moderate comorbidities. All participants were previous denture wearers who reported difficulties with the lower denture. All cases had unfavorable lower ridges (Cawood and Howell class IV or worse), which endorses the patient-perceived needs for more than conventional complete dentures. They were treated only after undergoing the same clinical examination and decision-making process expected in a standard dental office, the latter based on offering alternative treatment plans and discussing perceived needs and treatment limitations. Regarding treatment complexity, the attachments were similar; therefore, we expect that clinicians accustomed to work with overdentures to reach comparable outcomes.

The current study on implant-retained overdentures validates our previous model of satisfaction with the denture (de Souza et al. 2020) and shows that the additional retention obtained with implants preserved the model’s between-item correlation pattern. However, the results of the qualitative analysis showed that the ability to insert/remove the overdentures also carries great importance in terms of satisfaction, which was overlooked by the previous framework. Therefore, we propose that the ability to insert/remove the denture be added to the framework of satisfaction and the MDSQ, at least when used with overdenture wearers. Moreover, we found a relationship between aesthetics/chewing and comfort in this qualitative study of overdentures, where better aesthetics and chewing can lead to superior comfort. This causality can be added to our previous framework of denture satisfaction. Further quantitative and qualitative studies on conventional dentures are required to validate the proposed correlation.

Sample size can be pointed out as an important limitation of this study. Our current sample was able to detect a significant difference for the primary outcome (general satisfaction with SIMO). This implies that the NL attachment tends to be marginally more satisfying than the LC attachment. A similar finding was observed for a question on stability, serving as an explanation for greater satisfaction. Many questions reached no significance with narrow 95% CIs, representing a conclusive trend toward not rejecting null hypotheses. The latter was observed for OHRQoL in general, implying that using one attachment or the other will not interfere with the outcome. Therefore, we consider our sample size to be sufficient from a statistical standpoint, according to our research plan (i.e., to detect differences in terms of the primary outcome).

Other limitations include the follow-up time. The period after provision of each system suffices for significant retention loss in LC (Jabbour et al. 2014) but is of unlikely length for complications associated with other components. Moreover, a slightly longer period (i.e., 6 mo) could be expected to result in similar patient satisfaction with mandibular overdentures (de Souzaet al. 2015). Our study envisioned a longer follow-up (12 mo after crossover) to determine maintenance needs with NL and LC, which was halted by the COVID-19 pandemic. That would provide better data on clinical adversities. However, satisfaction and preferences are expected to remain the same given that patients receive proper prosthetic maintenance.

Potential limitations include the impossibility of blinding researchers and participants. This was tackled by 2 approaches: 1) avoiding any comment regarding the expected better performance of either attachment and not disclosing which of the tested systems each participant received (although they could see differences, participants were unable to determine which was the intervention or the control device); 2) even if the researcher providing prosthetic care could identify the attachments, data collection was performed by researchers who were unaware of what the participants were using during each follow-up. This implies that differences in patient satisfaction were led by the performance of each attachment rather than bias. Given that care providers cannot be blind, one could point to this as a potential source of bias. However, the similar clinical event rates with NL and LC suggest the opposite.

In summary, this study highlights that SIMOs retained by NL are more satisfying than those with LC if medium retentive inserts are used. Maintenance needs tended to be similar between the systems during the first 3 mo, a period during which attachments will undergo most of their wear-related changes (de Souza et al. 2015; Arnold et al. 2020; Wichmann et al. 2020). Further studies should elucidate whether differences remain the same over more extended periods, including maintenance needs. It is also plausible to expect a better result with NL for overdentures retained by ≥2 implants, but this should be confirmed by future clinical studies.

## Conclusions

The NL attachment system provides greater satisfaction with SIMOs than with the LC attachment system in edentulous elders during a 3-mo period. Reasons include better patient-reported denture stability combined with similar maintenance needs.

## Author Contributions

R.F. de Souza, contributed to conception and design, data acquisition, analysis, and interpretation, drafted and critically revised the manuscript; A.A. Jabbar, contributed to design, data acquisition, analysis, and interpretation, drafted and critically revised the manuscript; D. Jafarpour, contributed to design, data analysis and interpretation, drafted the manuscript; C. Bedos, contributed to design, data acquisition, analysis, and interpretation, critically revised the manuscript; S. Esfandiari, N.M. Makhoul, D. Dagdeviren, contributed to conception and design, data acquisition, critically revised the manuscript; S. Abi Nader, contributed to conception and design, data interpretation, critically revised the manuscript; J.S. Feine, contributed to conception and design, data analysis and interpretation, drafted and critically revised the manuscript. All authors gave final approval and agree to be accountable for all aspects of the work.

## Supplemental Material

sj-docx-1-jct-10.1177_23800844221124083 – Supplemental material for Single-Implant Overdentures Retained by a Novel Attachment: A Mixed Methods Crossover Randomized Clinical Trial Click here for additional data file. Supplemental material, sj-docx-1-jct-10.1177_23800844221124083 for Single-Implant Overdentures Retained by a Novel Attachment: A Mixed Methods Crossover Randomized Clinical Trial by R.F. de Souza, A.A. Jabbar, D. Jafarpour, C. Bedos, S. Esfandiari, N.M. Makhoul, D. Dagdeviren, S. Abi Nader and J.S. Feine in JDR Clinical & Translational Research
