# Supplementary material for: Single-Implant Overdentures Retained by a Novel Attachment: A Mixed Methods Crossover Randomized Clinical Trial
Source: JDR Clin Trans Res. 2022 Sep 20;9(1):27–41. doi: 10.1177/23800844221124083 (PMC10725123; doi:10.1177/23800844221124083)
Supplement: sj-docx-1-jct-10.1177_23800844221124083 – Supplemental material for Single-Implant Overdentures Retained by a Novel Attachment: A Mixed Methods Crossover Randomized Clinical Trial [file sj-docx-1-jct-10.1177_23800844221124083.docx]

**Appendix Table 1.** Baseline data, patient-reported outcomes.

| **Variable** | **First Attachment** | **Mean (SD)** | **Mean Difference** | **95% CI of the difference** |
| --- | --- | --- | --- | --- |
| **MDSQ Items** | | | | |
| **1. Ease of cleaning** | NL (Novaloc) | 8.9 (0.5) | -0.8 | -1.4 to -0.2 |
|  | LC (Locator) | 9.8 (0.3) |  |  |
| **2. General Satisfaction** | NL | 6.5 (3.9) | -2.7 | -7.5 to 2.2 |
|  | LO | 9.1 (0.5) |  |  |
| **3. Ability to speak** | NL | 7.0 (3.4) | -3.0 | -7.2 to 1.2 |
|  | LC | 10.0 (0.0) |  |  |
| **4. Comfort** | NL | 5.8 (3.6) | -3.2 | -7.6 to 1.1 |
|  | LC | 9.1 (0.8) |  |  |
| **5. Aesthetics** | NL | 7.6 (2.6) | -2.4 | -5.6 to 0.8 |
|  | LC | 9.9 (0.1) |  |  |
| **6. Stability** | NL | 3.9 (3.8) | -4.6 | -9.3 to 0.1 |
|  | LC | 8.5 (1.3) |  |  |
| **7. Ability to chew** | | | | |
| *7.1. Difficult to chew food in general?* | NL | 5.1 (3.3) | -3.8 | -7.9 to 0.2 |
|  | LC | 8.9 (0.5) |  |  |
| *7.2. Fresh white bread* | NL | 6.2 (3.6) | -3.4 | -7.9 to 1.0 |
|  | LC | 9.6 (0.4) |  |  |
| *7.3. Hard cheese* | NL | 5.2 (3.2) | **-4.1*** | **-8.0 to -0.1*** |
|  | LC | 9.3 (0.9) |  |  |
| *7.4. Raw Carrots* | NL | 3.1 (3.6) | -2.4 | -9.6 to 4.9 |
|  | LC | 5.5 (4.8) |  |  |
| *7.5. Dry salami* | NL | 4.3 (3.5) | -0.3 | -6.3 to 5.7 |
|  | LC | 4.6 (3.0) |  |  |
| *7.6. Sliced steak* | NL | 3.2 (3.6) | 1.1 | -6.4 to 8.5 |
|  | LC | 2.1 (2.7) |  |  |
| *7.7. Raw Apples* | NL | 2.9 (2.5) | -3.5 | -8.5 to 1.5 |
|  | LC | 6.4 (3.3) |  |  |
| *7.8. Lettuce* | NL | 4.1 (3.1) | -2.3 | -6.7 to 2.1 |
|  | LC | 6.4 (2.2) |  |  |
| **8. Function** | | | | |
| *8.1. Is your food well chewed before swallowing in general?* | NL | 4.6 (3.7) | -2.9 | -8.0 to 2.3 |
|  | LC | 7.5 (3.4) |  |  |
| *8.2. Fresh white bread* | NL | 6.4 (3.7) | -1.5 | -5.8 to 2.8 |
|  | LC | 7.9 (1.6) |  |  |
| *8.3. Hard cheese* | NL | 5.7 (3.4) | -1.3 | -6.0 to 3.4 |
|  | LC | 7.0 (2.3) |  |  |
| *8.4. Raw Carrots* | NL | 5.7 (3.4) | -2.0 | -9.1 to 5.2 |
|  | LC | 7.0 (2.3) |  |  |
| *8.5. Dry salami* | NL | 3.0 (3.7) | -1.7 | -8.4 to 5.1 |
|  | LC | 4.9 (4.5) |  |  |
| *8.6. Sliced steak* | NL | 3.3 (3.5) | -2.0 | -8.6 to 4.7 |
|  | LC | 5.0 (0.7) |  |  |
| *8.7. Raw Apples* | NL | 3.3 (3.5) | -0.7 | -7.8 to 6.4 |
|  | LC | 5.2 (4.1) |  |  |
| *8.8. Lettuce* | NL | 3.7 (3.3) | -2.6 | -8.0 to 2.7 |
|  | LC | 6.3 (3.5) |  |  |
| **9. Oral condition** | NL | 5.3 (3.7) | -3.9 | -8.0 to 0.2 |
|  | LC | - 1. (1.4) |  |  |
| **OHIP-EDENT** | | | | |
| **Total Score** | NL | 77 (24) | -18 | -51 to 15 |
|  | LC | 95 (21) |  |  |
